# ConnectedAlign: a PPI network alignment method for identifying conserved protein complexes across multiple species

**DOI:** 10.1186/s12859-018-2271-6

**Published:** 2018-08-13

**Authors:** Jianliang Gao, Bo Song, Xiaohua Hu, Fengxia Yan, Jianxin Wang

**Affiliations:** 1 School of Information Science and Engineering, Central South University, Changsha, 410083 China; 2 College of Computing & Informatics, Drexel University, Philadelphia, 19104 USA; 3 College of Liberal Arts and Sciences, National University of Defence Technology, Changsha, 410073 China

**Keywords:** Network alignment, Big data, Graph data analysis

## Abstract

**Background:**

In bioinformatics, network alignment algorithms have been applied to protein-protein interaction (PPI) networks to discover evolutionary conserved substructures at the system level. However, most previous methods aim to maximize the similarity of aligned proteins in pairwise networks, while concerning little about the feature of connectivity in these substructures, such as the protein complexes.

**Results:**

In this paper, we identify the problem of finding conserved protein complexes, which requires the aligned proteins in a PPI network to form a connected subnetwork. By taking the feature of connectivity into consideration, we propose ConnectedAlign, an efficient method to find conserved protein complexes from multiple PPI networks. The proposed method improves the coverage significantly without compromising of the consistency in the aligned results. In this way, the knowledge of protein complexes in well-studied species can be extended to that of poor-studied species.

**Conclusions:**

We conducted extensive experiments on real PPI networks of four species, including human, yeast, fruit fly and worm. The experimental results demonstrate dominant benefits of the proposed method in finding protein complexes across multiple species.

## Background

A protein complex is a bimolecular that contains a number of proteins interacting with each other to perform different cellular functions which is described in many prior works such as the work proposed by Hu at al. in [1]. The identification of protein complexes in a protein-protein interaction (PPI) network [2] can, therefore, lead to a better understanding of the roles of such a network in different cellular systems. It is for this reason that the protein complex identification problem has received a lot of attentions, and a considerable number of techniques and algorithms have been proposed to address such problem. Graph structure is widely adopted in many applications [3, 4]. By representing a PPI network as a graph [5], whose vertices represent proteins and edges as interactions between proteins, these algorithms are able to identify clusters in single PPI network based on different graph properties [6]. For example, an uncertain graph model based method is proposed to detect protein complex from a PPI network [7]. To identify protein complexes, previous works proposed to consider not just topological but also biological information in the network [1]. However, they all focused on finding protein complexes in a single PPI network, and finding conserved protein complexes from multiple PPI networks still remains challenging.

Network alignment provides a possible way to identify protein complexes from multiple PPI networks [8]. Conserving functional and topological features are two goals for network alignment. Functional module represents a collection of molecular interactions that work together to achieve a particular functional objective in a biological process, while topological module represents locally dense neighborhoods in a PPI network [9]. Network alignment can be categorized into two classes: global alignment and local alignment. Global alignment [10] finds overall best functional orthologs among entire PPI networks, while local alignment identify smaller conserved subnetworks in part of the networks [11]. In the context of local alignment, when a given small network is aligned with large networks, the problem can be projected as network query problem. In this paper, we concern more on the local alignment, which is more related to our problem.

**Fig. 1:**
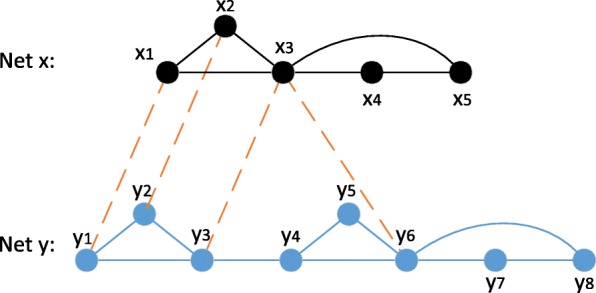
Disconnected sub-network problem. Proteins are represented by vertices, PPIs by solid lines, and links between bipartite graphs by dashed lines. Traditional pairwise local alignment might miss the desired protein complex. For example, *x*_1_,*x*_2_ are aligned to *y*_1_ and *y*_2_, but *x*_3_ might be aligned to *y*_6_ when maximizing the vertex similarity score, which results in disconnected substructure

Traditional pairwise network alignment detects functional orthologs of proteins in PPI networks by maximizing the similarity between proteins, while ignoring the subnetwork structure of protein complex. Therefore, the disconnected subnetwork problem might be caused when applying those methods to identify conserved protein complexes. For example, in Fig. 1, there are two PPI networks Net *x* and Net *y*. When aligning complex (*x*_1_,*x*_2_,*x*_3_) in Net *x* to Net *y*, protein *x*_1_ and *x*_2_ are aligned with *y*_1_ and *y*_2_. But only maximizing pairwise similarity of proteins might lead *x*_3_ to be aligned with *y*_6_, which results in disconnected subnetwork in the alignment and doesn’t meet well with the requirement of protein complex.

**Fig. 2:**
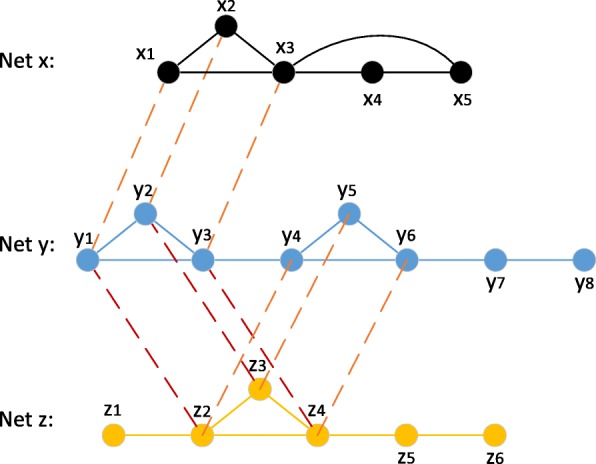
Inconsistency problem. Applying traditional pairwise local alignment in multiple alignment might miss the desired protein complex. For example, when (*x*_1_,*x*_2_,*x*_3_) is aligned to (*y*_1_,*y*_2_,*y*_3_), (*z*_2_,*z*_3_,*z*_4_) might be aligned to (*y*_4_,*y*_5_,*y*_6_) while (*y*_1_,*y*_2_,*y*_3_) is the more consistent alignment. Then, inconsistency arises in Net *y*

Aligning multiple networks promises additional insights into the protein complexes as well as the knowledge-transfer across multiple species. However the alignment of multiple PPI networks has additional challenges. For example, if directly applying the methods of pairwise network alignment to the multiple network alignment, inconsistency problem might be caused. For example, as shown in Fig. 2, the substructure (*x*_1_,*x*_2_,*x*_3_) in Net *x* is aligned with (*y*_1_,*y*_2_,*y*_3_) in Net *y*. When they are expected to be further aligned with the (*z*_2_,*z*_3_,*z*_4_) in Net *z* from consistent perspective, (*y*_4_,*y*_5_,*y*_6_) might be the best alignment instead if it was a pairwise alignment between Net *y* and Net *z*. However, since the goal of multiple network alignment is to find conserved protein complexes across all PPI networks, (*y*_1_,*y*_2_,*y*_3_) should be a better result.

In this paper, we propose a new approach to find conserved protein complexes by network alignment. The main contributions are as follows:

- We identify the problem of finding conserved protein complex via aligning multiple PPI networks. In this way, the knowledge of protein complexes in well-studied species can be extended to that of many poor-studied species.
- We propose an efficient method to find conserved protein complexes from multiple PPI networks. In this method, we take the feature of subnetwork connections into consideration, which improves the coverage significantly without compromising the consistency of aligned results.
- Extensive experiments are conducted on the PPI networks of four species including human, yeast, fruit fly and worm. These results in terms of coverage and consistency illustrate the dominant benefit of the proposed method in finding protein complexes across species.

## Method

### Problem definition

#### Definition 1.

Target network: A PPI network *G*_*t*_=(*V*_*t*_,*E*_*t*_) is called target network if the given protein complexes to be aligned belong to *G*_*t*_, where *V*_*t*_ is the set of proteins and *E*_*t*_ is the set of interactions between them.

The knowledge such as protein complexes of a target network can be extended to other PPI networks via network alignment. We define the other PPI networks as *aligned networks*.

#### Definition 2.

Aligned networks: Let *G*={*G*_*i*_}(1≤*i*≤*ξ*) be the set of aligned networks, where *ξ* is the number of PPI networks to be aligned with target network. *G*_*i*_=(*V*_*i*_,*E*_*i*_)(1≤*i*≤*ξ*) is the *i*^*th*^ PPI network to be aligned, where *V*_*i*_, *E*_*i*_ are the sets of proteins and their interactions.

Given target network, aligned networks and protein complexes of target network, we define the input of the problem as follows.

**Input:** (1) The set of aligned networks *G*={*G*_*i*_,1≤*i*≤*ξ*}, where *ξ* is the number of aligned networks. (2) The set of well studied protein complexes in target network *G*_*t*_: *S*={*S*_1_,*S*_2_,...*S*_*ζ*_}, where *ζ* is the number of protein complexes to be aligned.

Then the alignment result as the output is defined as follows.

**Output:** Without loss of generality, for any protein complex *M*_0_, *M*_0_∈*S*, the alignment result is a matchset *M*={*M*_1_,*M*_2_,…,*M*_*ξ*_} consists of a set of *ξ* subnetworks, where *M*_*k*_⊆*G*_*k*_, 1≤*k*≤*ξ*, *G*_*k*_∈*G*, which satisfies: (1) any *M*_*k*_⊆*G*_*k*_ is a connected subnetwork of *G*_*k*_; (2) maximizing the similarity score of {*M*_0_,*M*_1_,*M*_2_,…,*M*_*ξ*_}.

With the definitions and notations above, our algorithm of finding protein complexes across multiple PPI networks via network alignment mainly follows two procedures: assigning scores to proteins according to both biological and structural features, and then heuristically selecting proteins that form connected subnetwork in each PPI network which finally achieves optimized total score for multiple PPI networks.

### Scoring strategy of network alignment

Overall, we utilize both the biological similarity between proteins and the topological structure to assign scores on subnetworks for subsequent heuristic selections of proteins. Formally, given a protein complex of target network *M*_0_⊆*G*_*t*_, its match result {*M*_1_,*M*_2_,…,*M*_*ξ*_} in aligned networks, where *M*_*k*_⊆*G*_*k*_, is assigned with a real-valued score *Φ*:

1 $$ \Phi = \sum\limits_{k \in \{1,\ldots,\xi \}} \sum\limits_{v_{j} \in V_{M_{k}}} \left(\alpha * \delta_{bio}(v_{j}) + (1-\alpha) * \delta_{topo}(v_{j}) \right)  $$

where *ξ* is the number of PPI networks, $V_{M_{k}}$ is the set of proteins in *M*_*k*_, *α* is a coefficient to trade off biological and topological scores, *δ*_*bio*_ and *δ*_*topo*_ are the biological and topological scores respectively. In the following, we will describe the details of determining the *δ*_*bio*_ and *δ*_*topo*_.

Assume *M*_*k*_⊆*G*_*k*_ is the current subnetwork to be assigned a score, where *G*_*k*_, 1≤*k*≤*ξ*, is the current aligned network. At each time, choose another PPI network denoted as *G*_*h*_, (*h*≠*k*)∧(1≤*h*≤*ξ*), then *G*_*t*_,*G*_*k*_,*G*_*h*_ construct a group of triple networks. Denote *M*_*h*_⊆*G*_*h*_ as subnetwork of *G*_*h*_ to align with *M*_0_. For every *h*, we calculate score for the proteins in *G*_*k*_ in the triple networks.

**Fig. 3:**
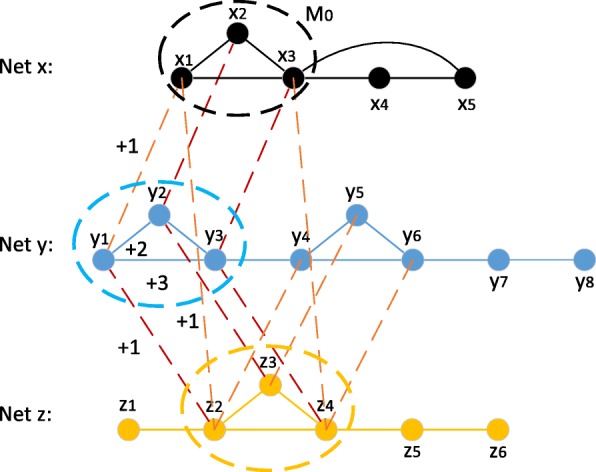
Illustration of assigning scores. Net *x* is the target network, and *M*_0_ is the given protein complex. Net *y* is an aligned network. Taking *y*_1_ as example, its scores $(\delta _{bio}^{1}, \delta _{bio}^{2}, \delta _{bio}^{3}, \delta _{topo}^{1}, \delta _{topo}^{2})$ are 1, 1, 1, 2, 3, respectively

We use Fig. 3 as an example to show the method of assigning scores, where *M*_0_ is the target subnetwork in target network Net *x* consisting of (*x*_1_,*x*_2_,*x*_3_), *M*_*k*_ is the subnetwork in aligned network Net *y* to be assigned scores consisting of (*y*_1_,*y*_2_,*y*_3_). And the subnetwork of (*z*_2_,*z*_3_,*z*_4_) in aligned network Net *z* is to be aligned with *M*_0_.

#### Definition 3.

Link: If a pair of proteins (*u*,*v*) comes from different PPI networks, and *u*,*v* are sequence similar, then (*u*,*v*) is called a link.

Sequence similarity [12] can be obtained with the BLASTP method [13]. We connect a dashed line to denote a link in this paper.

#### Definition 4.

Thread: If triple proteins (*u*,*v*,*t*) comes from three different PPI networks, and there exist links between (*u*,*v*), (*u*,*t*) and (*v*,*t*) at the same time. Then they form a thread.

The biological score of a protein consists of: (1) the number of links with the subnetwork *M*_0_, (2) the number of links with the subnetwork *M*_*h*_, and (3) the number of threads among these three subnetworks which contain the current protein. We denote these three scores as $\delta _{bio}^{1}$, $\delta _{bio}^{2}$, $\delta _{bio}^{3}$. Taking *y*_1_ in Fig. 3 as example, there are links (*y*_1_,*x*_1_), (*y*_1_,*z*_2_) and thread (*y*_1_,*x*_1_,*z*_2_). Therefore, $\delta _{bio}^{1}$, $\delta _{bio}^{2}$, $\delta _{bio}^{3}$ of vertex are all “1". To avoid excessive influence of one factor, we adopt a transform techniques by multiplying a coefficient. The biological score of a protein *u* is:

2 $$ \delta_{bio}(u) = {\left(\delta_{bio}^{1}\right)}^{\frac{1}{\lambda}} + {\left(\delta_{bio}^{2}\right)}^{\frac{1}{\lambda}} + {\left(\delta_{bio}^{3}\right)}^{\frac{1}{\lambda}}  $$

where $\delta _{bio}^{1}$, $\delta _{bio}^{2}$, $\delta _{bio}^{3}$ are the numbers of links with *M*_0_, *M*_*h*_ and the number of threads respectively. *λ*(*λ*>1) is the parameter of transform.

#### Definition 5.

Component: a connected graph *G*_*c*_=(*V*_*c*_,*E*_*c*_) is a component of subnetwork *M*_*k*_ if *G*_*c*_⊆*M*_*k*_.

The topological score of a vertex consists of (1) the degree of current vertex; (2) the size of the maximal component that includes the current vertex. As the same with biological score, we adopt a transform techniques by multiplying a coefficient. The topological score of a vertex *u* is:

3 $$ \delta_{topo}(u) = {\left(\delta_{topo}^{1}\right)}^{\frac{1}{\omega}} + {\left(\delta_{topo}^{2}\right)}^{\frac{1}{\omega}}  $$

where $\delta _{topo}^{1}$ is *u*’s degree in its subnetwork, and $\delta _{topo}^{2}$ is the size of the maximal component that includes *u*. *ω* is a parameter of transform. In our method, *ω*>1.


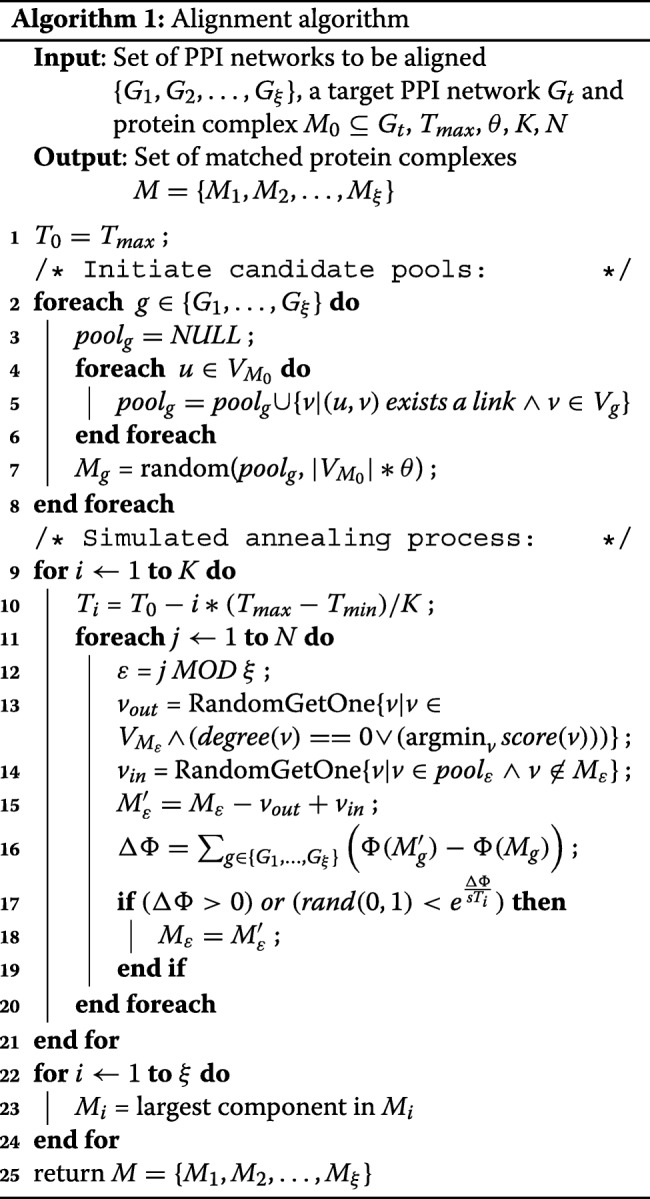


### Alignment algorithm

Given the multiple PPI networks and target protein complex from the target PPI network, the alignment process is shown in Algorithm 1, which It mainly includes:

(1) Generate initial candidate pools.

Only those proteins that have links with given protein complex can be selected as candidate proteins since links represent the biological similarity between proteins across PPI networks according to Definition 3. For each aligned network *G*_*i*_1≤*i*≤*ξ*, we construct a pool for a given protein complex *M*_0_, where *M*_0_∈*G*_*t*_. All vertices in *G*_*i*_ are put into the pool of *G*_*i*_ if they have links with any vertex in *M*_0_, as shown in Line 5 of Algorithm 1. Then, the initial subnetworks *M* are selected randomly from the pools.

(2) Simulated annealing process.

Simulated annealing process adopts iteration method for global optimal solution. In each loop, a protein from the candidate pool is chosen randomly to be determined as aligned protein in the corresponding PPI network (Line 14 of Algorithm 1). On the other hand, there are two kinds of proteins that could be moved out from the current alignment solution (Line 13 of Algorithm 1). The first kind is the protein whose score is the lowest in the current solution: $ \{ v | v \in V_{M_{\varepsilon }} \wedge {\text{argmin}}_{v}score(\textit {v}) \} $. The other kind is the protein whose corresponding vertex in the current subnetwork is not connected with other vertices, i.e., its degree is zero. As shown in Line 16–19 of Algorithm 1, if the new candidate solution achieves higher score, it will take place the previous solution. If not, it still has chance to replace the prior solution with a probability of $\left (rand(0,1)<e^{\frac {\Delta \Phi }{sT_{i}}}\right)$, where $e^{\frac {\Delta \Phi }{sT_{i}}}$ returns the selection threshold for the selection of simulated annealing process. Finally, the algorithm returns the best solution as the alignment of protein complexes *M*={*M*_1_,*M*_2_,…,*M*_*ξ*_}.

## Results and discussion

In this section, we evaluate the performance of our method through extensive experiments. We compare our method to LocalAli [14] since LocalAli is the most recent local alignment method for PPI networks. We measure the coverage and consistency of the alignment networks.

### Dataset and experimental setup

Real-world PPI networks of four species are used in our experiments, including Homo sapiens (human), Dorsophila melanogaster (fruit fly), Caenorhabditis elegans (worm) and Saccharomyces cerevisiae (yeast) [15]. The detailed numbers of proteins and interactions for each species are listed in the Table 1.

**Table 1:** Proteins and interactions of four species

PPI networks	# Proteins	# Interactions
A:Human(H.sapiens)	11,258	47,031
B:Worm(C.elegans)	9,302	15,669
C:Fruit Fly(D.melanogaster)	8,725	27,053
D:Yeast(S.cerevisiae)	5,494	54,163

We also obtained the corresponding sequences of all proteins from manually annotated and reviewed database UniProtKB/Swiss-Prot [16] for calculating pairwise protein similarity, i.e., e-value, by conducting BLASTP 2.3.0 (downloaded from the NCBI BLAST [17]) and setting *e*^−7^ as the e-value cutoff, to select the potential homologous proteins across different species. The corresponding Gene Ontology (GO) annotations of the proteins are collected from the Uniprot-GOA database for the alignment evaluations.

As human and yeast are the two best studied species [18], we build data sets by assigning them alternatively as the target PPI network for the alignment, and choose two from the rest of our collected PPI networks as aligned networks. There are total of six datasets generated, with each dataset as a group of multiple PPI networks to perform alignment. The composition of the six datasets are listed in Table 2.

**Table 2:** Datasets composition

Datasets	Target species A	Aligned species B	Aligned species C
D1	A:Human	B:Worm	C:Fruit Fly
D2	A:Human	B:Worm	D:Yeast
D3	A:Human	C:Fruit Fly	B:Worm
D4	D:Yeast	B:Worm	A:Human
D5	D:Yeast	B:Worm	C:Fruit Fly
D6	D:Yeast	C:Fruit Fly	A:Human

With most local alignment algorithms that are pairwise, LocalAli [14] is one of the few most recent local alignment approaches. In LocalAli, a framework is proposed to reconstruct the evolution history of conserved modules based on a maximum-parsimony evolutionary model. LocalAli aims to identify functionally conserved modules from multiple biological networks, which is able to be used as a comparison method to our proposed algorithm. We run LocalAli with its default parameters on the six datasets in Table 2 to obtain target protein complexes, by retrieving every matchset in its results and obtain whose proteins form a component in the target network. The components from the target network are used as the input of our algorithm. In the experiment, we set the parameters *α*=0.5,*θ*=1.1,*K*=20,*N*=100,*T*_*max*_=100,*λ*=4.5,*ω*=3. The results are compared with LocalAli in terms of coverage and consistence.

### Coverage

A larger and denser connected component can give more insight of common topology of the network and it could be more biologically significant. The coverage analyzes the numbers of proteins in the aligned subnetworks from each aligned PPI networks with the given motifs in the target network.

As shown in Table 3, We compare our algorithm with LocalAli [14] on the six datasets, where D1 ∼D3 are assigning human PPI network as the target network and D4 ∼ D6 get the yeast as the target network. For each dataset, since we utilize the largest component in the according target PPI network from the LocalAli as our target protein complex for alignment, the average number of proteins in every target network are all the same to that of the LocalAli, i.e., ratio is 100% for the target network. The ratio is the result obtained by dividing the average size of protein complexes of our proposed method by that of LocalAli. As in the aligned networks, our method can generate larger sizes of aligned protein complexes than that of the LocalAli among all datasets. One exception is in the dataset D3, where two method obtained equal coverage in one of the aligned networks, while obtaining much higher coverage in the other aligned networks. Similar situation exist in dataset D6. In dataset D1, D2, and D4, our algorithm achieves significantly higher coverage in all aligned networks, with the largest one has nearly 248% coverage to the LocalAli.

**Table 3:** Comparison of coverage

Dataset	PPI network	Average size of protein complexes		Ratio
		Proposed	LocalAli [14]	
D1	target network A	7.05	7.05	100.00%
	aligned network B	4.73	3.64	129.95%
	aligned network C	5.54	3.07	180.46%
D2	target network A	8.56	8.56	100.00%
	aligned network B	4.99	2.75	181.45%
	aligned network D	9.89	3.99	247.87%
D3	target network A	6.86	6.86	100.00%
	aligned network C	3.07	3.07	100.00%
	aligned network B	7.86	5.79	135.75%
D4	target network D	7.74	7.74	100.00%
	aligned network B	7.09	3.47	204.32%
	aligned network A	8.82	7.79	113.22%
D5	target network D	5.05	5.05	100.00%
	aligned network B	4.28	3.95	108.35%
	aligned network C	3.55	3.32	106.93%
D6	target network D	5.81	5.81	100.00%
	aligned network B	4.31	4.13	104.36%
	aligned network A	6.88	5.75	119.65%

The ratio is the result obtained by dividing the average size of protein complexes of our proposed method by that of LocalAli

### Consistency

The calculation of the consistency utilizes the Gene Ontology (GO) annotations associated to each of the proteins, with three basic types of ontologies describing biological properties: biological process (BP), molecular function (MF) and cellular component (CC) [19]. It is assumed that proteins with more similar GO annotations are more functionally coherent [20]. We calculate and analyze such functional similarity by the fraction of aligned proteins that share same GO annotations. The larger the fraction, the more biological significance the alignment has.

The consistency, specifically measured by the mean entropy (ME) and mean normalized entropy (MNE), serves as a specificity metric to measure the quality of alignment. To calculate ME, we first obtain the entropy *E*(*M*) of a matchset M, i.e. the protein complexes aligned to one protein complex in the target species among all participated PPI networks, with following formulation:

4 $$ E(M)=E(v_{1},v_{2},\ldots v_{n}) = - \sum\limits_{i=1}^{d}p_{i} \times log (p_{i})  $$

where *p*_*i*_ is the fraction of all proteins in the matchset *M* with the annotation *GO*_*i*_, and d represents the total number of different GO terms in *M*. Thus the aligned matchset with more consistency will have lower entropy. The ME of the matchset is then calculated by averaging the entropies of all matchsets generated from the alignment to all the protein complexes in the target species, and the lower the ME of the alignment results, the higher consistency a method performs, indicating a better biological quality.

Similar to ME, for the MNE, we first calculate the normalized entropy NE(M) for a matchset as:

5 $$ NE(M)=NE(v_{1},v_{2},\ldots v_{n}) = -\frac{1}{log d} \sum\limits_{i=1}^{d}p_{i} \times logp_{i}  $$

where *p*_*i*_ and *d* have the same interpretation of those in the *E*(*M*). The MNE of the alignment results is then computed by calculating the average of the normalized entropy of all matchsets with their size. The lower MNE, the better functional consistency an alignment method achieves.

The comparison of consistency between the results from LocalAli and our algorithm is shown in Table 4. The ratio is the result obtained by dividing the *ME* or *MNE* of our proposed method by that of LocalAli then subtracting one. We can observe that in D1, D4, D5 and D6, our method generates aligned protein complexes with slightly higher ME and MNE than that of the LocalAli, where the ratio of the consistency less to LocalAli range from 0.76 to 6.48%. Meanwhile, we achieve higher ME and MNE than LocalAli in D2 and D3, with 8.12% better consistency at most.

**Table 4:** Comparison of consistency

Dataset	Metrics	Consistency		Ratio
		Proposed	LocalAli [14]	
D1	ME	18.72	17.58	6.48%
	MNE	4.19	4.06	3.20%
D2	ME	33.76	35.97	-6.14%
	MNE	6.34	6.90	-8.12%
D3	ME	18.50	18.63	-0.70%
	MNE	4.27	4.30	-0.70%
D4	ME	35.70	34.59	3.21%
	MNE	6.62	6.57	0.76%
D5	ME	11.1	10.82	2.59%
	MNE	2.90	2.87	1.05%
D6	ME	17.72	17.44	1.61%
	MNE	4.13	4.09	0.98%

The ratio is the result obtained by dividing the *ME* or *MNE* of our proposed method by that of LocalAli then subtracting one

For PPI network alignment, it is more important to achieve the alignment of functional modules than the alignment of proteins alone. The proposed ConnectedAlign achieves this goal without losing the consistence and coverage. In the future, the genome information could be used for biological network alignment [21].

## Conclusion

In this paper, we proposed a novel approach to identify conserved protein complexes across different species. Given target protein complexes in the target network, the proposed method can find conserved protein complexes in multiple aligned PPI networks. Since we take the biological feature and topological feature into consideration, including subnetwork connectivity, our method achieves higher coverage significantly, and keeps stable consistence compared with previous network alignment method. The experimental results demonstrate the significant benefits of our proposed alignment method.
